# Analysis of research on gamification, critical thinking and academic performance: A systematic review in the context of higher education

**DOI:** 10.12688/f1000research.164232.1

**Published:** 2025-09-12

**Authors:** Natalie Margarita Gamarra-Vargas, Ricardo Fernando Cosio Borda, Percy Hugo Quispe-Farfán, Fabiola Gabriela Bosmans Flores, Liz Maribel Robladillo-Bravo

**Affiliations:** 1 Graduate School, Universidad Peruana de Ciencias Aplicadas, Lima District, Lima Region, 15076, Peru; 2 Faculty of Administration, Finance and Economic Sciences, Universidad EAN, Bogotá, Bogota, 111321, Colombia; 3 Business Faculty, Universidad Peruana de Ciencias Aplicadas, Lima District, Lima Region, 15023, Peru; 4 Universidad Autónoma de Ica, Chincha Alta, Ica, 11701, Peru

**Keywords:** gamification, critical thinking, academic performance, higher education

## Abstract

Gamification has become relevant in the field of higher education, facilitating the teaching and learning process. There are studies that show a positive relationship between gamification and academic performance. A systematic literature review was developed, applying the prism method and using three databases: Scopus, WOS and Scielo. The results indicate a notorious orientation of the studies towards quantitative research. Ninety-two percent of the selected documents have students as their population, while 6% are oriented to teachers and 2% to both. In the more in-depth results, it can be demonstrated that gamification is linked to the generation of critical thinking and the improvement of academic performance in the context of higher education. The analysis of theoretical structures reveals that the most relevant approaches for the study of gamification are self-determination and gamified learning. It is also evident that there is a positive relationship between gamification and motivation in learning processes, which allows encouraging the development of critical thinking and the improvement of academic performance.

## 1. Introduction

Gamification is a technique that has taken relevance in the field of higher education, being consistent with the nature of human beings and societies, where games have an important role in cultural development. In this sense, there are studies that refer to the importance of games in the different areas of human development, summarizing it under the concept of Homo Ludens ( Bozkurt and Durak, 2018; Caillois, 2001; Huizinga, 1938; Krath et al., 2021). In short, the aforementioned is not alien to the educational field.

In relation to the context presented previously, referring to gamification necessarily implies the use or application of game elements, but in a context that is not playful. Therefore, the purpose is not to entertain but to generate people’s involvement in other activities that may be seen as complex, unattractive or even challenging ( Gao, 2024; Krath et al., 2021). Following this logic, learning is a process that can often be seen as challenging, which is why gamification has been acquired as an element that contributes to generate motivation among the actors in this scenario and there are several studies that support it ( Bai et al., 2020; Gao, 2024; Zainuddin et al., 2020).

In the educational context, gamification is considered a technique that employs game dynamics in the aforementioned environment, allowing to obtain a better academic result by the people being taught, increasing students’ concentration, generating motivation and involving learners in the learning process more efficiently. In short, it allows the training experience to be enhanced and learning to be intensified ( Ismail and Mohammad, 2017; Johns, 2015; Licorish et al., 2018; Magadán and Rivas, 2022a, 2022b; Robson et al., 2015; Pineda-Martínez et al., 2023; Vives et al., 2022).

It is important to point out that in literature there are authors who understand gamification as a synonym for learning based on games and serious games ( Coutinho and Lencastre, 2019; Flores-Bueno et al., 2021; Lozada and Betancur, 2017; Pascuas et al., 2017). There are positions that indicate that the concept of gamification is like an umbrella that contains everything linked to gaming in education, but that there are its particularities and differences ( Anastasiadis et al., 2018; Alsawaier, 2018; Flores-Bueno et al., 2021; Limaymanta et al., 2020).

Referring to gamification and its popularization in the field of education is linked to the importance of motivation in the learning process. There are studies that support the positive relationship between gamification and motivation, hence its relevance in the educational field and the need to keep the student motivated in the learning process ( Ekici, 2021; Krath et al., 2021; Koivisto and Hamari, 2019; Sailer and Homner, 2020). Also, from a psychophysiological perspective there are elements to take into consideration such as satisfaction, positive attitude towards the game, enjoyment, immersion and flow ( Ab Jalil et al., 2020; Boyle et al., 2016; Connolly et al., 2012; Ekici, 2021; Koivisto and Hamari, 2019; Lamb et al., 2018; Vlachopoulos and Makri, 2017).

Regarding the theoretical bases that support gamification, there are several contributions, among which the use of the following stand out: self-determination theory, relevance theory, the transtheoretical model of behavior change, cognitive load theory, individual and social constructivism, the technology acceptance model, social cognitive theory (self-efficacy as the central axis) and Landers’ gamified learning ( Bandura, 1997; Banfield and Wilkerson, 2014; Davis, 1989; Keller, 1987; Krath et al., 2021; Landers, 2014; Piaget; 1977; Polo-Peña et al., 2021; Prochaska and Velicer, 1997; Ryan and Deci, 2000; Seaborn and Fels, 2015; Sweller, 1988; Vygotsky and Cole, 1978; Wilson, 1973). In this sense, gamification is studied from different theoretical approaches to understand its application and the benefits it generates.

Performing an initial review in the SCOPUS database, taking as an initial search element, the equation “gamificación OR gamification”, limiting the results to a time range from 2020 to 2024, considering the thematic area of social sciences and considering the criterion “all open access”; 712 documents were obtained (number valid as of November 21, 2024). With the aforementioned information base, we proceeded to perform an analysis in Bibliometrix, to identify some general information that would contribute to generate a first scope of the progress of gamification studies and those clusters of knowledge most relevant to gamification.

**Figure 1.**
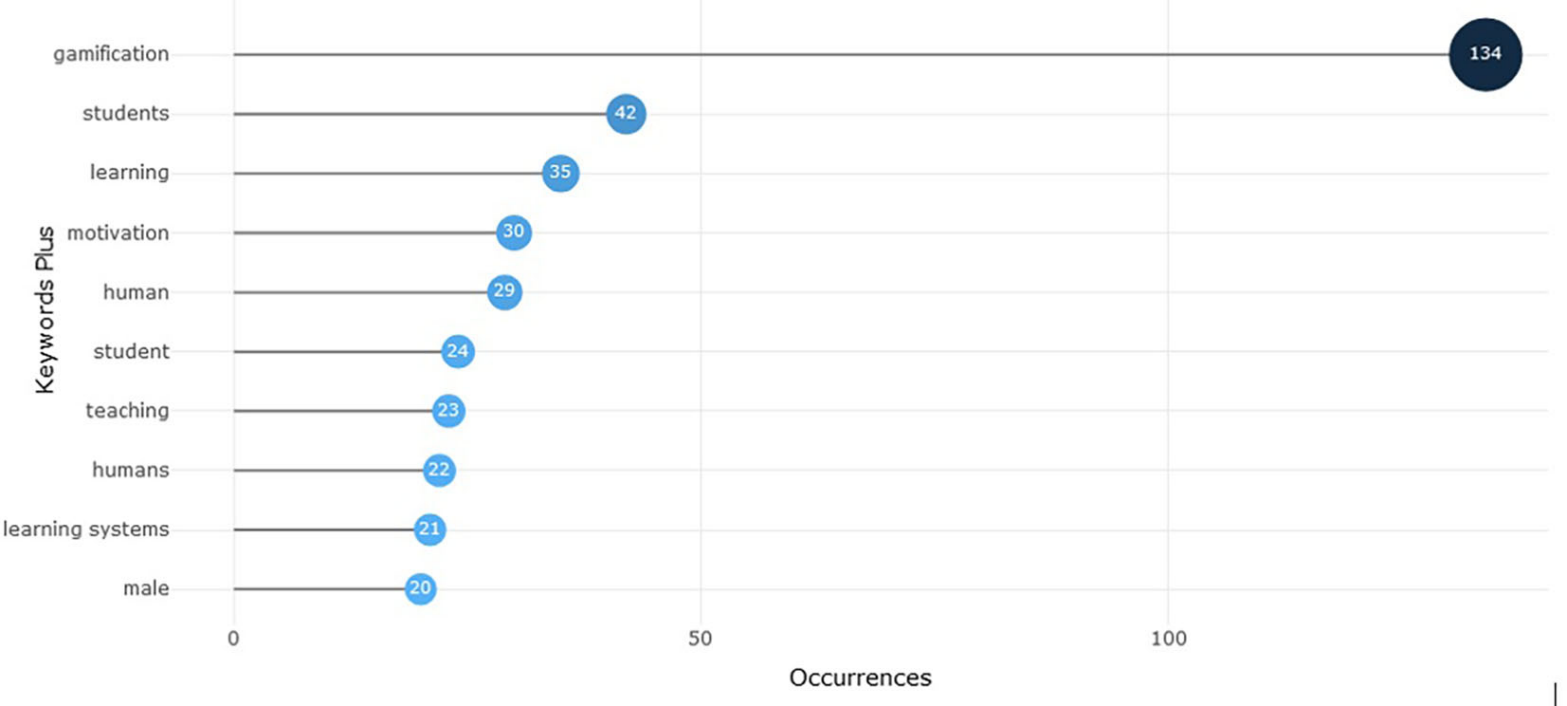
Most relevant words about gamification scientific paper. Note. The graph presents the words with the highest number of occurrences or appearances in the documents extracted from SCOPUS on gamification. Taken from Bibliometrix on November 21, 2024.

**Figure 2.**
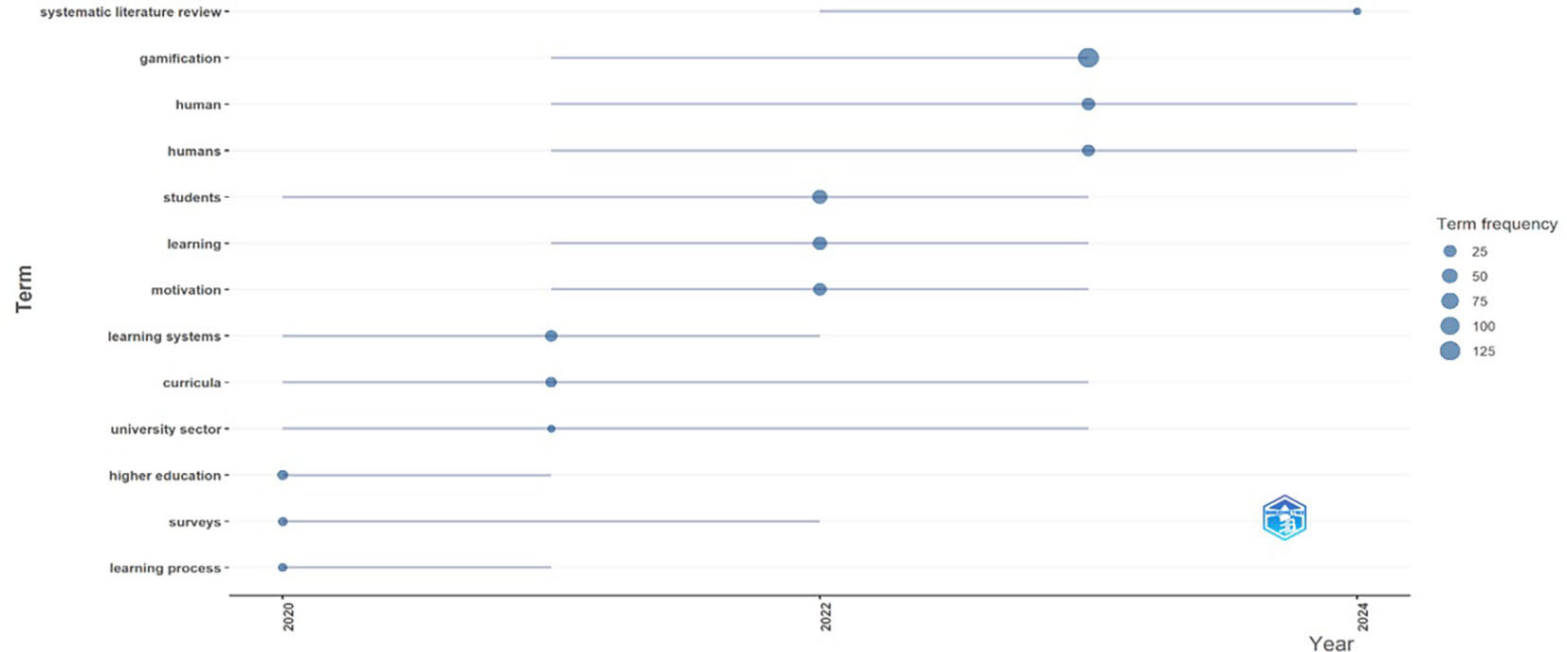
Trending topics according to the analysis of scientific papers on gamification. Note. The graph presents the topics that are trending in the documents extracted from SCOPUS on gamification. Taken from Bibliometrix on November 21, 2024.

According to Figure 1, it can be identified that the most recurrent words in scientific papers on gamification are linked to the field of education, such as “learning”, “student”, “teaching” and “learning systems”. This reality is important to support the relevance of the present text, which orients the analysis from the context of education. Likewise, this aspect is ratified in Figure 2 that shows the topics that are trending according to the scientific documents analyzed in Bibliometrix, among which some new terms such as “university sector” and “higher education” emerge, which also support the orientation of the present study towards higher education. Similarly, the term “systematic literature review” as an emerging topic shows the growing interest of the scientific community in conducting studies of this type, such as the one proposed in this document.

**Figure 3.**
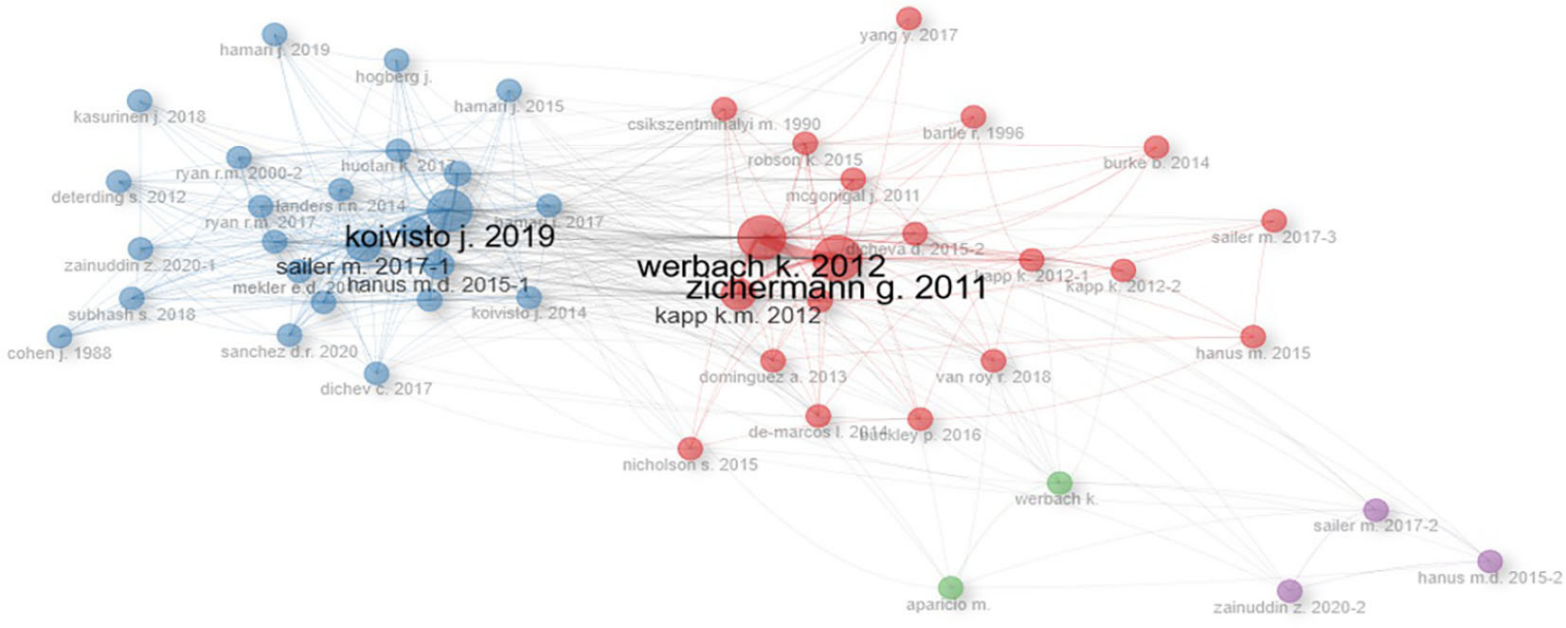
Co-citations network of scientific papers on. Note. The graph presents the co-citations network of papers extracted from SCOPUS on gamification. Taken from Bibliometrix on November 21, 2024.

Regarding Figure 3, the co-citation network can be observed, where the most cited authors in the scientific papers on gamification that were analyzed and also the interaction between them are shown. This graph is also relevant to highlight that the analysis carried out in the first paragraphs of this paper contains authors that appear in the network, such as Koivisto, Hamari, Saller and others. The latter highlights the relevance of the literature review conducted in this first part of the introduction.

**Figure 4.**
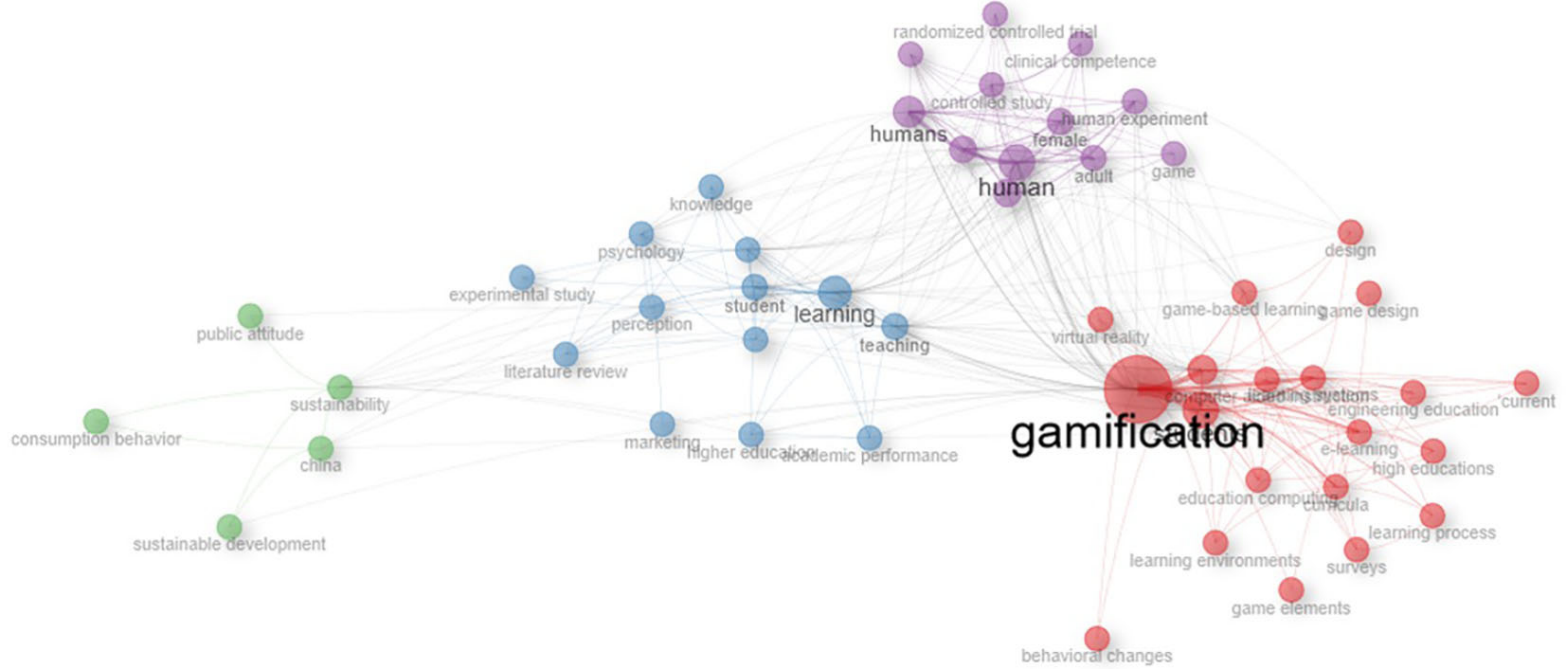
Co-occurrence network of concepts of gamification scientific papers. Note. The graph presents the network between the main concepts according to the appearances or occurrences in the documents extracted from SCOPUS on gamification. Taken from Bibliometrix on November 21, 2024.

**Figure 5.**
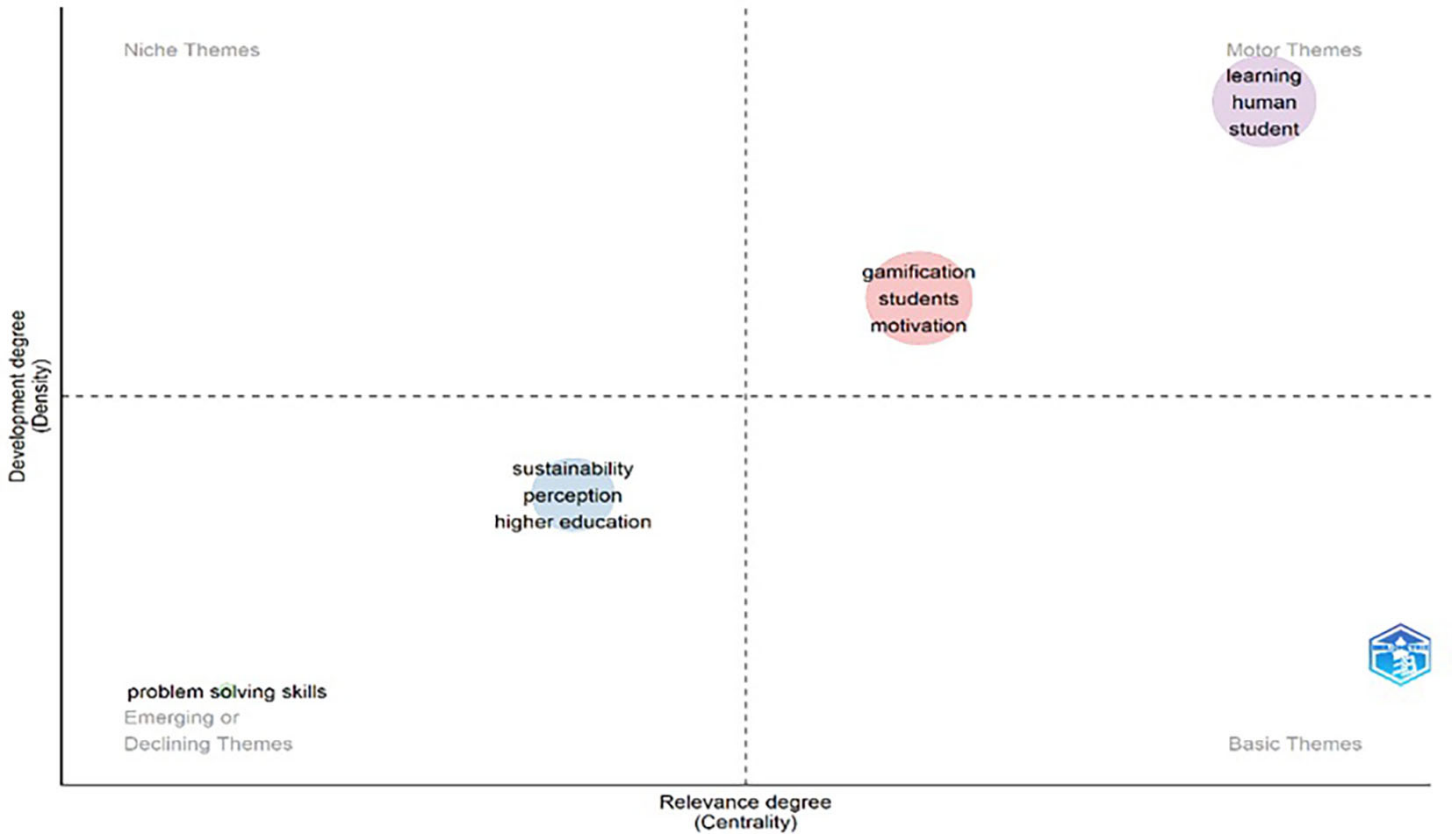
Topic map of gamification scientific papers. Note. The graph presents the main topics addressed in the papers extracted from SCOPUS on gamification Taken from Bibliometrix on November 21, 2024.

Figures 4 and 5 contain important elements that highlight the need to approach gamification studies from an educational perspective. In the network of co-occurrence of concepts, one can visualize a cluster located in the lower right part (red color) and another in the middle part of the graph (blue color) where there are several concepts on learning, teaching and education that are addressed. Similarly, the map of themes ( Figure 5) ends up closing the idea of the relevance of approaching the study of gamification from education at a higher level, since there is medium and high relevance, with a density of equal characteristics.

The previous analysis allows us to pose the following research question: How has the scientific literature on gamification, critical thinking and academic performance evolved in the context of higher education? In this sense, the main objective is to analyze the evolution of the scientific literature on gamification, critical thinking and academic performance in the context of higher education. The specific objectives are: 1) to determine the search criteria of scientific research on gamification, critical thinking and academic performance in the context of higher education; 2) to describe the search results of scientific research on gamification, critical thinking and academic performance in the context of higher education; and, 3) to analyze the theoretical structures and main findings of the scientific literature on gamification, critical thinking and academic performance in the context of higher education.

## 2. Methods

Given the growth of papers interested in gamification, and with the aim of finding the main findings and research gaps related to it, a systematic review was conducted using a systematic mapping approach based on the PRISMA (Preferred Reporting Items for Systematic Reviews and Meta-Analyses) methodology, which transparently documents why the review was conducted, the process of searching and compiling the sample articles, and what findings were found ( Haddaway et al., 2022). This allows for a reproducible description, critical review, and synthesis of the findings for future research.

**Figure 6.**
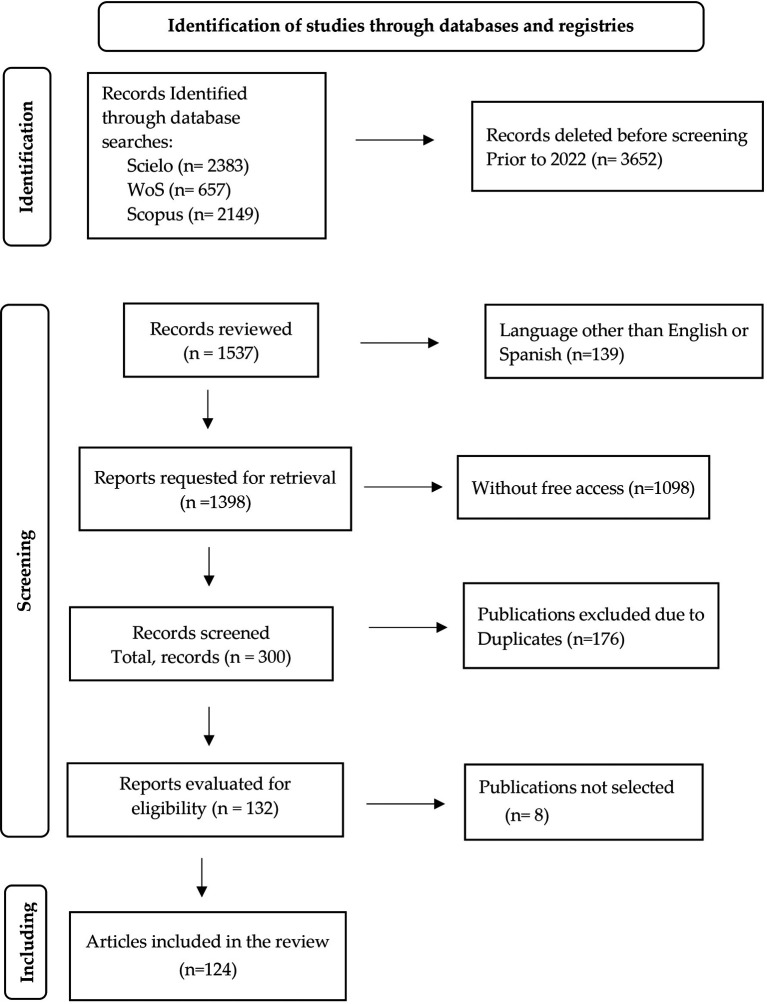
Process based on PRISMA method. Note. The PRISMA flowchart is presented.

The main databases were searched for: Web of Science (last search date: August 27, 2024), Scopus (last search date: August 27, 2024) and Scielo (last search date: August 27, 2024). Only studies in English and Spanish were included in these databases. The selection procedure is included in the PRISMA flow in Figure 6. The keywords or equations used in Table 1 are gamification, critical thinking and academic performance. Since the present paper intends to focus on the development of gamification in university students that enhances critical thinking and improves the academic performance possessed by the students, the period limitation of the previous 3 years was established.

**Table 1.** Equations for searches.

N°	Equations
1	Gamification AND higher AND education
2	Gamification AND university
3	Critical AND thinking AND higher AND education
4	Critical AND thinking AND university
5	Academic AND performance AND higher AND education
6	Academic AND performance AND university
7	Gamification AND critical AND thinking
8	Gamification AND academic AND performance
9	Critical AND thinking AND academic AND performance
10	Gamification AND critical AND thinking AND academic AND performance

**Table 2.** Selected articles.

Database	Articles
WOS	Aibar-Almazán et al. (2024), Álvarez-Huerta et al. (2022), Double et al. (2023), Du & Zhang (2022), Hilario et al. (2022), Kabilan et al. (2023), Lee et al. (2023a), Li et al. (2022), Liu & Pásztor (2022), Lyons et al. (2023), Mehrpour et al. (2023), Navarro-Espinosa et al. (2022), Ng & Lo (2022), Orhan (2024), Pagán et al. (2023), Parody et al. (2022), Pineda-Martínez et al. (2023), Redondo-Rodríguez et al. (2022), Ren & Barrett (2023), Rivas et al. (2022), Rivas et al. (2023b), Ruiz-Rojas et al. (2024), Sousa-Vieira (2023), Teng & Yue (2023), Van et al. (2023), Zembylas (2024), Zhang et al. (2023).
SCOPUS	Abanto-Ramirez et al. (2024), Abd Halim et al. (2024), Abu et al. (2024), Alhamuddin et al. (2023), Almulla (2023), Alrashed et al. (2023), Álvarez-Huerta et al. (2024), Andrés (2022), Andreucci-Annunziata et al. (2023), Angelelli et al. (2023), Bekbayeva et al. (2022), Cao et al. (2022), Das et al. (2024), Du & Zhang (2022), Dumitru & Minciu (2023), Dumitru et al. (2023), Elvsaas et al. (2023), Fraguas-Sánchez et al. (2022), Garcés-Fuenmayor et al. (2023), González-Limón et al. (2022), González et al. (2022), Guerrero-Alcedo et al. (2022), Gutiérrez-Pingo et al. (2023), Guzmán-Valenzuela et al. (2023), Hell et al. (2022), Ivarson et al. (2024), Jabali et al. (2024), Kamran et al. (2022), Kleemola et al. (2023), Kubiatko et al. (2022), Kuzina et al. (2022), Lee et al. (2023b), Low et al. (2024), Lv et al. (2022), Lyons et al. (2023), Magadán-Díaz & Rivas-Garcia (2022a), Martínez et al. (2022), Mejía & Lara (2022), Morote & Hernández-Hernández (2024), Mumpuni et al. (2023), Muñoz & Gasca-Hurtado (2023), Muslimin & Abidin (2023), Najafi et al. (2022), Núñez-Pacheco et al. (2023), Onieva & Rojas (2023), Orhan (2023), Ostendorf & Thoma (2022), Othman et al. (2023), Palacios-Núñez et al. (2023), Paľová & Vejačka (2022), Panmei & Waluyo (2022), Peña-González et al. (2023), Pozo-Sánchez et al. (2022), Priyaadharshini & Maiti (2023), Rebelo et al. (2023), Rivas et al. (2023a), Rodríguez-Iglesias et al. (2022), Romsi et al. (2024), Russo et al. (2023), Salarvand & Mousavi (2022), Sánchez et al. (2023), Sawiji et al. (2024), Schendel et al. (2023), Shakurnia et al. (2022), Suartama et al. (2023), Sümer & Aydın (2022), Supnoon & Chonchaiya (2024), Suryadi et al. (2023), Vachova et al. (2023), Valencia-Rodríguez et al. (2022), Varela-Tapia et al. (2022), Vázquez-Parra et al. (2023), Vendrell-Morancho (2024), Vendrell-Morancho et al. (2024), Vergara et al. (2023), Witarsa & Muhammad (2023).
SCIELO	Aboderin & Havenga (2024), Acosta-Yela et al. (2022), Adam & Berg (2022), Aldás & López (2023), Araiza-Vazquez et al. (2023), Berrío-Quispe et al. (2024), Borrego et al. (2022), Hernández & Mayorga (2022), Magadán-Díaz & Rivas-García (2022b), Medel-San Elías et al. (2022), Medel-San Elías et al. (2023), Muñoz & Gasca-Hurtado (2023), Navarro-Sempere et al. (2022), Ojeda et al. (2022), Olivo García et al. (2023), Papageorgiou (2023), Ramiro Miranda et al. (2022), Sarabia-Guevara & Bowen-Mendoza (2023), Vera-Sagredo et al. (2023), Zepeda Hurtado et al. (2022).

First, articles were selected that were published between 2022 and 2024 in peer-reviewed scientific journals, that are open access, full text, written in Spanish and/or English. Likewise, they should be qualitative, quantitative or mixed studies, addressing the established subject matter. The theoretical studies are excluded; articles with a language other than Spanish and English and those published prior to 2022 will not be considered. In addition, articles that do not contain studies whose participants were not university students during the selection phase will be discarded.

After replication, 132 articles were identified, 124 studies met the requirements. Based on the studies focused on gamification in university students that enhance critical thinking and improve the academic performance of students.

Finally, with the final corpus of articles, a manual review was carried out based on an Excel table systematically organized by columns to extract information from each work, considering the mapping and research questions. Regarding data processing, the information was synthesized to obtain an updated study of the current state of the field of study and the research gaps detected. It should be noted that all authors of this document were reviewers of each record and document retrieved. Likewise, all authors were responsible for reviewing the collected data.

For the systematic review, this document used the PRISMA flowchart which is a fundamental tool to visually represent the study selection process in a systematic review or meta-analysis ( Haddaway et al., 2022). A detailed table to be used for each section of the PRISMA flowchart is presented in Figure 6 and the final list of selected papers is shown in Table 2.

## 3. Results

**Figure 7.**
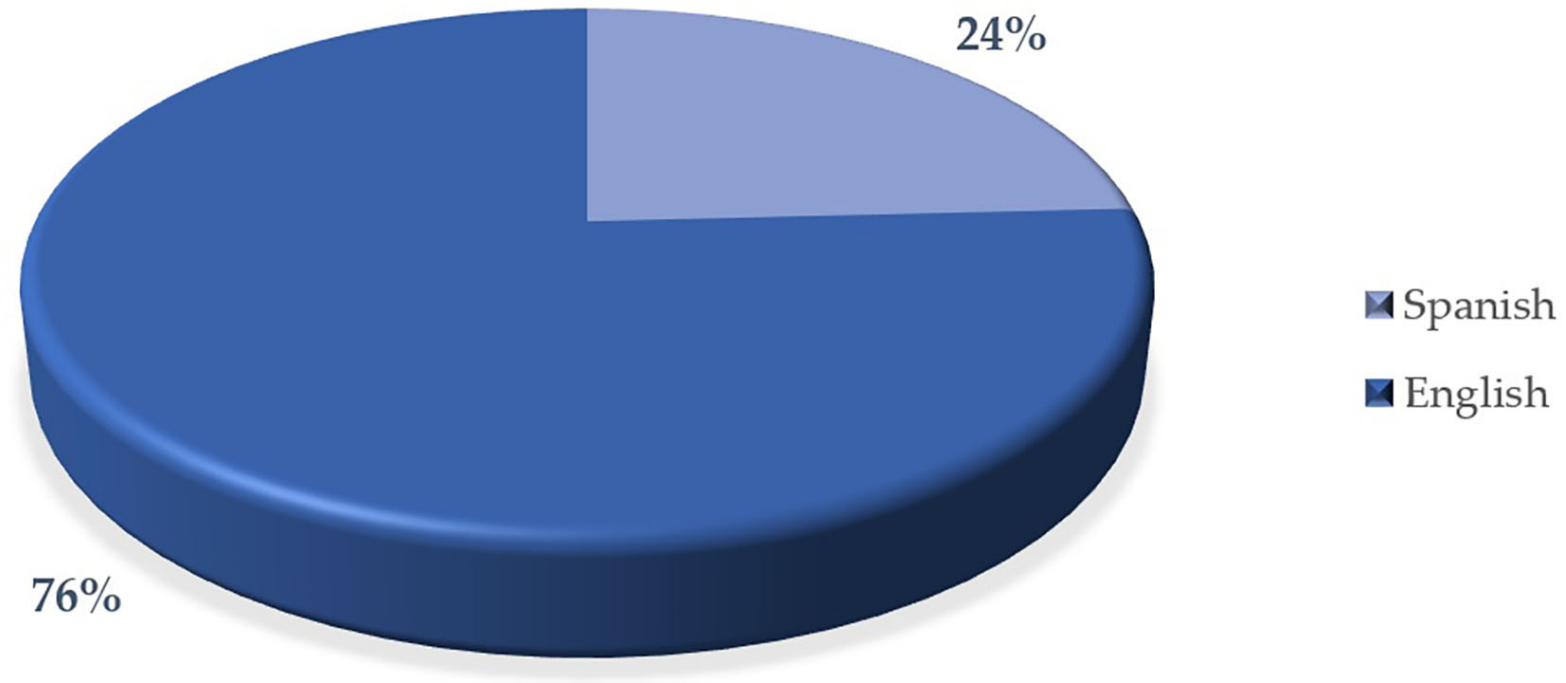
Articles by language. Note. The graph presents the papers by language.

Figure 7 indicates that there is a higher proportion of articles in English (76%) compared to articles in Spanish (24%). This suggests that the predominant language in the articles analyzed is English. The difference in the proportion is significant, which could reflect the importance or focus on English in scientific or academic publishing in interest.

This result could be related to the global trend of using English as the main language for scientific dissemination, which facilitates greater visibility and international impact of research.

**Figure 8.**
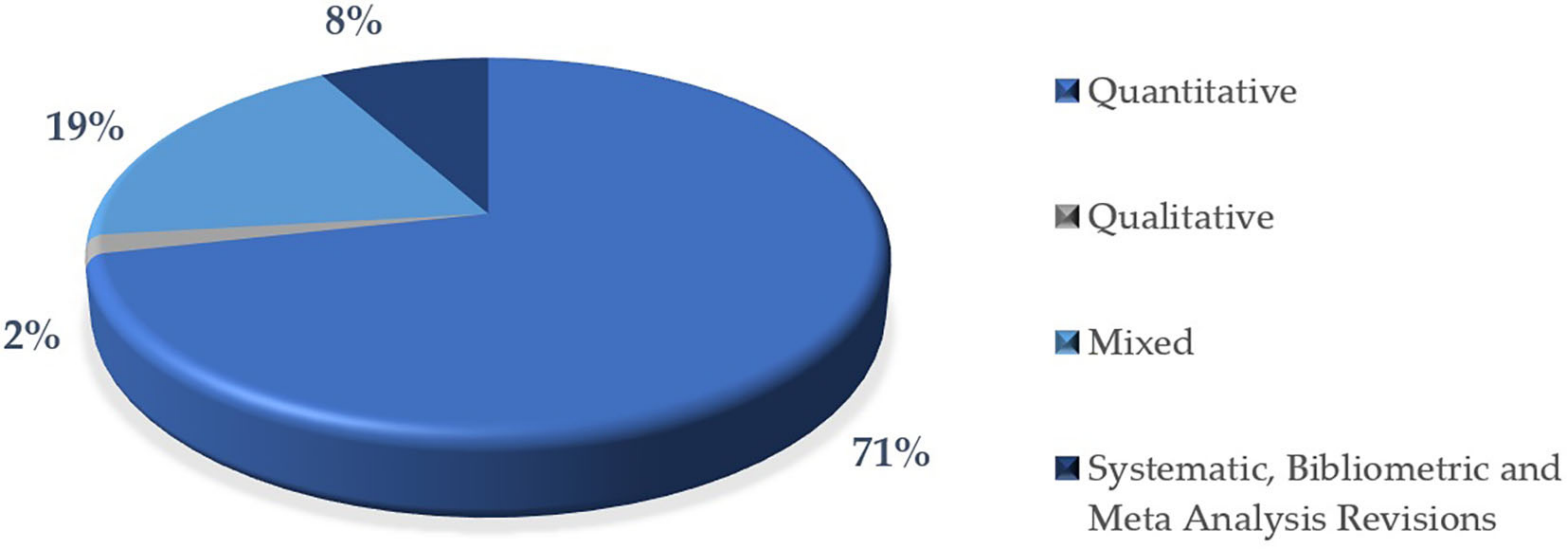
Articles according to the methodological approach of the research. Note. The graph presents the papers according to the methodological approach of the research.

In this research, most of the articles use a quantitative methodology 71%, indicating an approach based on the collection and analysis of numerical data. This high percentage reflects a tendency towards objectivity and measurement in research, which are predominant characteristics in many scientific areas. This information is represented graphically in Figure 8.

On the other hand, a significant proportion of the articles combine quantitative and qualitative methods, in this case 19%. This mixed approach is useful for integrating numerical analyses with contextual interpretations, providing a more complete perspective on the phenomena studied.

In contrast, a very low percentage used exclusively qualitative methodologies for this study is 2%, which may suggest a lower prevalence of studies focused on narrative, content analysis or exploration methods.

Systematic reviews, bibliometric reviews and meta-analyses represent 8%. This category shows how these techniques focus on rigorously analyzing, synthesizing and evaluating existing knowledge in an area, helping to identify trends, gaps and patterns in scientific literature.

Finally, this distribution pattern could be indicative of a disciplinary bias towards the natural, social or applied sciences, where the quantitative method predominates.

**Figure 9.**
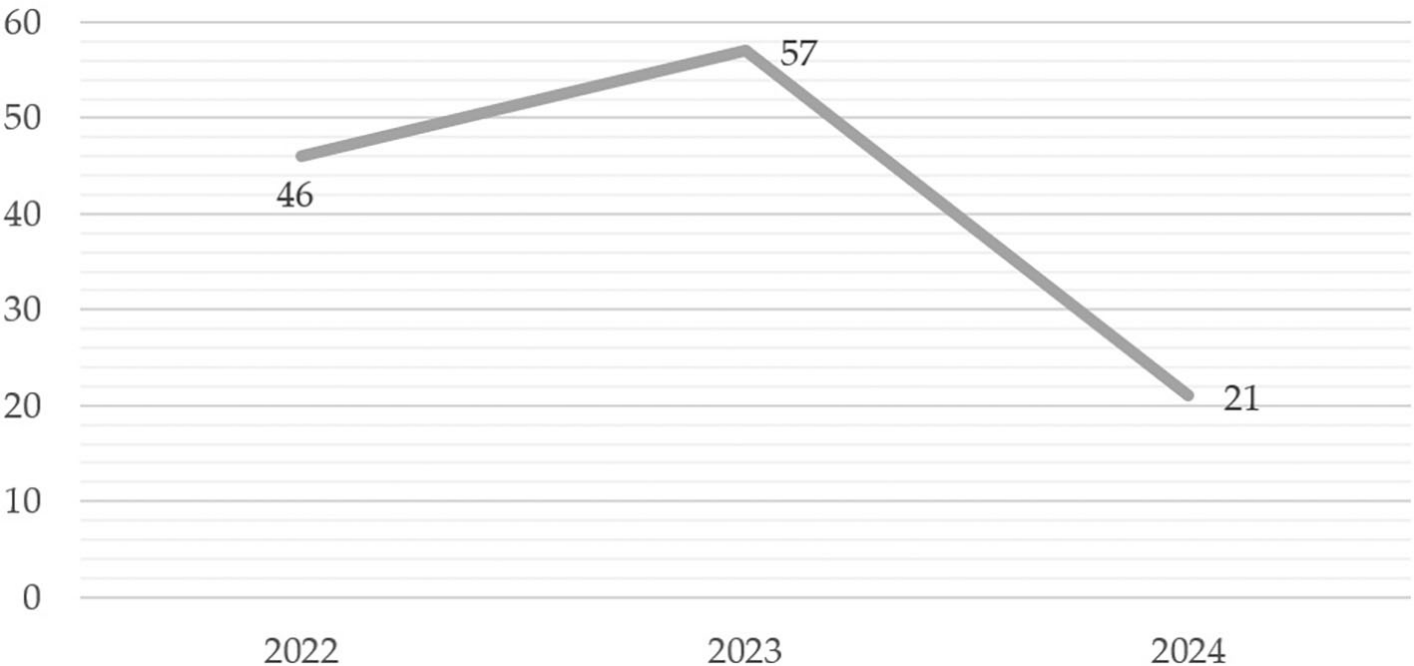
Evolution of the number of articles published per year (2022-2024). Note. The graph presents the evolution of the number of papers published per year (2022-2024).

The analysis of scientific production over the last few years reveals significant trends in the number of publications, making it possible to identify patterns of growth and decline that could be associated with contextual and temporal factors. A summary of the data for the years 2022, 2023 and 2024 is presented in Figure 9, highlighting the main changes in each period.

In 2022, with 46 published articles, there is a moderate level of scientific production, which serves as a starting point for analyzing the trend in subsequent years. On the other hand, in 2023 there is a notable increase in the number of publications, reaching 57 articles, representing an increase of 24% over the previous year. This boom could reflect a greater interest around study on “gamification” or be related to relevant events that boosted research. However, in 2024, the picture changes drastically, as the number of publications drops to 21 articles, representing a 63% decrease compared to 2023. Also, this decline can be interpreted in two ways. This could indicate a possible real reduction in research activity, explained by changes in scientific priorities, less funding, or difficulties in the publication process. Finally, the fluctuations observed reflect not only the dynamism of scientific production, but also the impact of external and internal factors on academic research.

**Figure 10.**
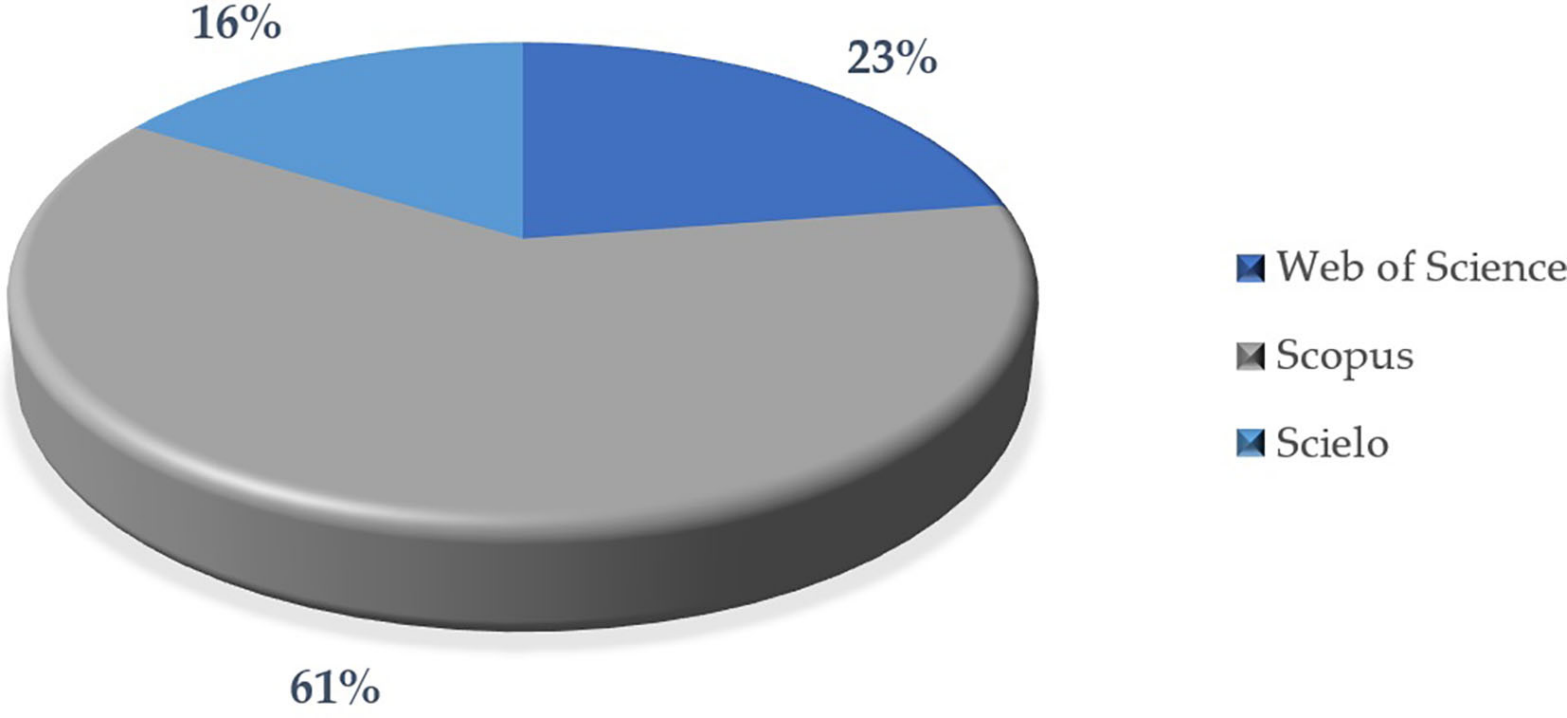
Articles according to database. Note. The graph presents the papers according to database.

The analysis in Figure 10 examines the distribution of scientific articles in three important databases: Scopus, Web of Science and Scielo. The comparison highlights the characteristics and scope of each database, as well as their relevance in the selected sample.

First, most of the articles are indexed in Scopus (61%), indicating that this database is the main source for publications in this sample. Scopus is recognized for its broad scope and rigor in the indexing of scientific journals, which could explain its predominance.

On the other hand, a significant proportion of the articles (23%) are indicated in Web of Science, which is also a high quality and prestigious database. Although it has a lower participation than Scopus, it is still a relevant source in the sample.

In contrast, a smaller proportion of articles (16%) are published in journals indexed in Scielo. This could be because Scielo has a more regional focus (mainly in Latin America), while Scopus and Web of Science tend to have a more global scope.

Finally, the predominance of Scopus could be related to the global focus of the research and the search for greater visibility and academic impact. Likewise, the participation of Web of Science reinforces the relevance of high quality and prestigious journals in the selection of the publications analyzed. Finally, the lower representation of Scielo could suggest that these publications have a more localized or regional focus, which may limit their inclusion in other more global databases.

**Figure 11.**
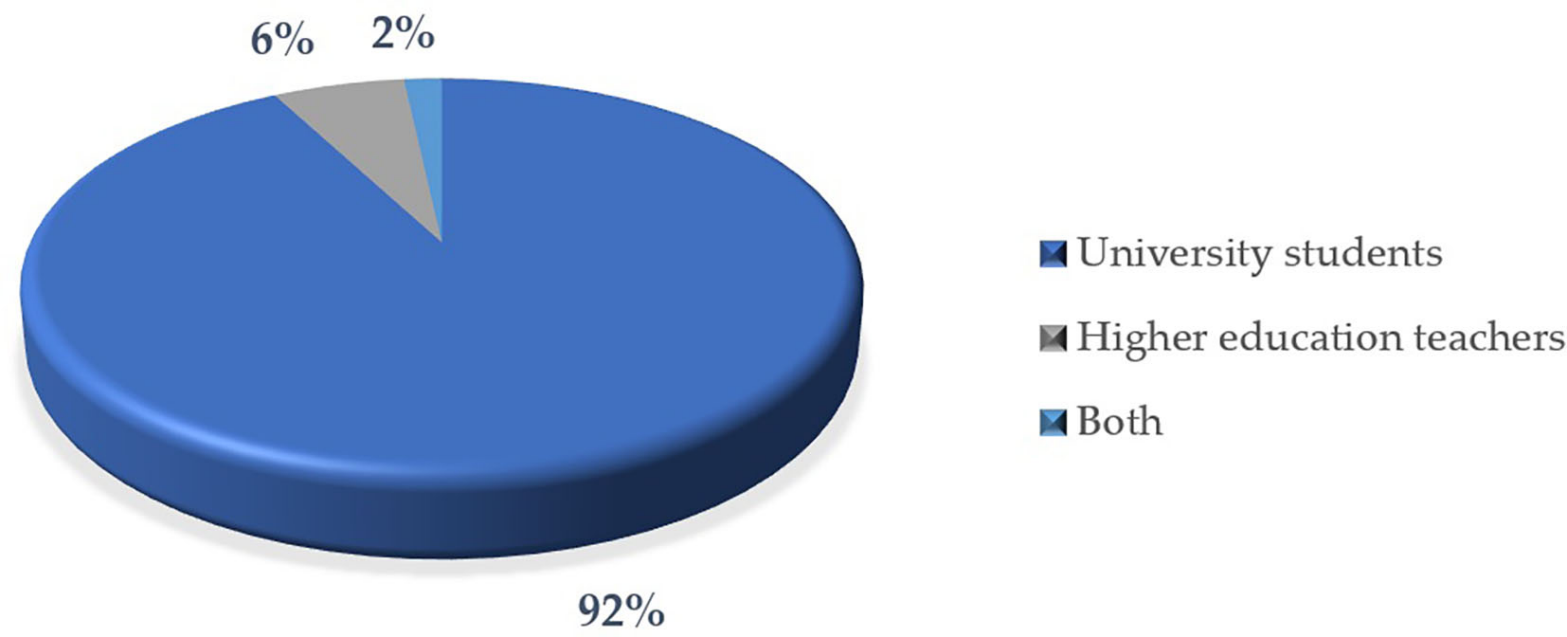
Items by study population. Note. The graph presents the papers by study population.

Analysis of the populations studied in the education articles reveals significant trends with respect to the interest groups within this field ( Figure 11). The following is a breakdown of the main approaches, highlighting priorities and possible areas of opportunity in the research conducted:

Firstly, students represent 92% of the populations studied, being the main group on which most of the articles are focused due to the research variables which in this case are “gamification”, “education”, “university”, “critical thinking”. This fact reflects a clear interest in analyzing educational processes, behaviors, skills or learning outcomes in this group. On the other hand, a small percentage of the articles, equivalent to 6%, focus on educators. This, however, could be evidence of an interest in exploring the perspectives, competencies or pedagogical practices of teachers in comparison with students.

Furthermore, only 2% of the studies include both groups, students and educators, simultaneously. This marginal percentage indicates a lower tendency toward comprehensive analyses that consider the interaction between these two populations.

Consequently, the predominance of students as the focus of research highlights an interest centered on the impact of educational practices on the main recipients of educational systems. Likewise, the low percentage of studies focused on educators represents an opportunity to deepen knowledge about the role and needs of teachers in learning environments.

In relation to the previous paragraphs, the limited representation of studies that jointly address students and educators points to an under-explored area of research. This aspect is relevant, especially in studies that seek to analyze the joint dynamics between teaching and learning.

## 4. Discussion

Performing a deeper analysis of the selected documents following the PRISMA method, the following results can be identified in the case of the articles extracted from WOS.

**Figure 12.**
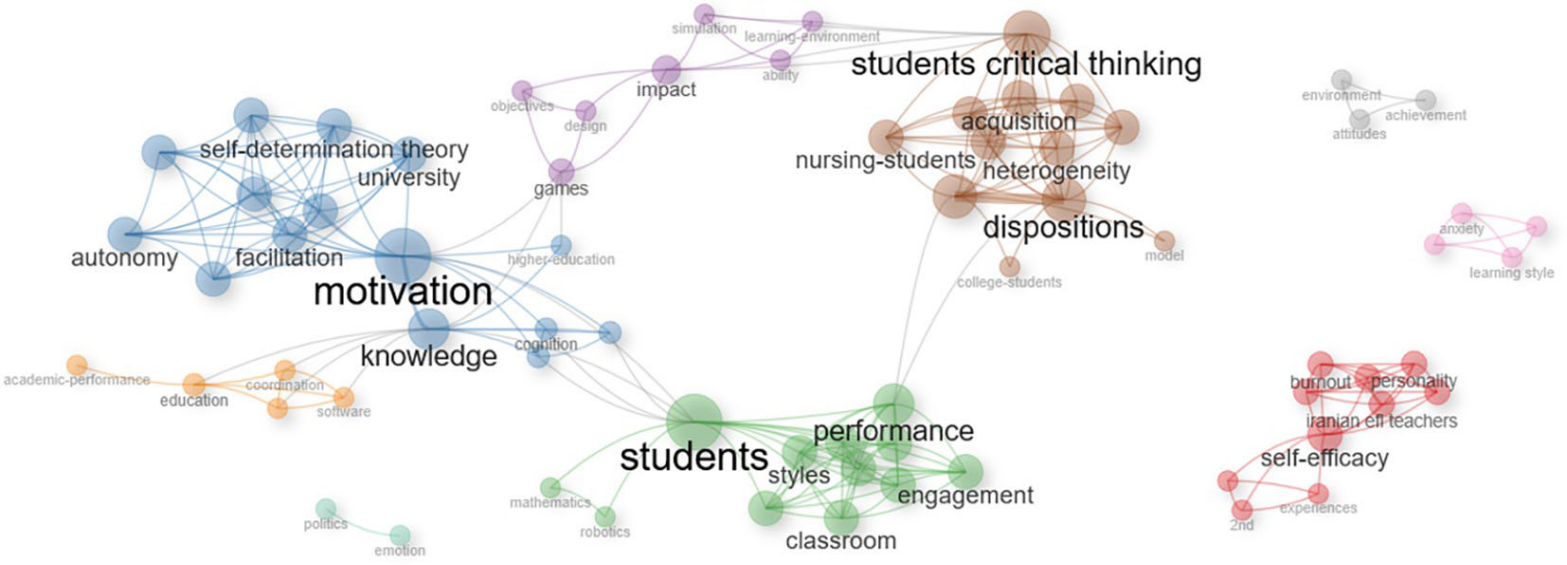
Network based on the thematic map of scientific papers extracted from WOS. Note. The graph presents the network based on the thematic map of scientific papers extracted from Web of Science.

An interesting relationship can be identified between the whole blue sector, which is centralized by motivation, with the purple one oriented to games and the whole brown block corresponding to critical thinking. In this sense, it is possible to affirm that according to the selected literature there is an interesting link between the motivation generated by games in learning environments with the development of critical thinking in students. This is closely consistent with the main theories that are addressed in the various gamification studies, such as the case of self-determination theory, which is highlighted in the network shown in Figure 12. This relevance lies in the fact that motivation is the center of human behavior according to this postulate, which clearly expressed implies that a subject motivated through gamified activities in learning environments can develop critical thinking ( Gao, 2024; Ryan and Deci, 2000).

On the other hand, another association can be identified between the purple, blue and green areas, which implies that transferring game mechanics to the educational environment contributes to generating motivation in students and favors academic performance. The above is in line with what is expressed in Landers’ gamified learning theory that highlights the benefits of this technique ( Landers, 2014) being one of them also proposed by the literature analysis shown in the previous network, where academic performance can be favored by the application of gamified activities during the learning process.

Another important aspect to highlight is the red network that is isolated from the other segments. In this sense, it can be noted that there is interest in conducting studies linked to the social cognitive theory that has self-efficacy as its central axis ( Bandura, 1997; Banfield and Wilkerson, 2014). However, an interesting area for future research is identified, where the relationship of self-efficacy with motivation, critical thinking and academic performance can be analyzed.

The network represents with sufficient clarity the orientation of the selected research. In the center of the whole graph are the students, to whom the gamification activities in learning environments are directed (red color block), which generates motivation and engagement in the learning process (purple block), developing skills that promote critical thinking in students (green and blue networks).

The aforementioned is closely related to what is expressed in the theory and previous studies analyzed in the introduction section. It is relevant to highlight that motivation continues to be a recurrent element in the selected studies, which also coincides with the network shown in the WOS documents. It can be stated according to what is shown in the network and contrasting with the studies and theories shown in the first paragraphs of this text that there are studies that argue and evidence the positive relationship between gamification and motivation in the learning process ( Ekici, 2021; Krath et al., 2021; Koivisto and Hamari, 2019; Sailer and Homner, 2020).

Similarly, the relationship shown by the network is sustained in a highly relevant fact indicated by the theories applied to study gamification. This comprises psychophysiological elements linked to satisfaction, positive attitude towards the game, enjoyment, immersion and flow ( Ab Jalil et al., 2020; Boyle et al., 2016; Connolly et al., 2012; Ekici, 2021; Koivisto and Hamari, 2019; Lamb et al., 2018; Vlachopoulos and Makri, 2017); elements that generate the application of gamification in learning processes.

**Figure 13.**
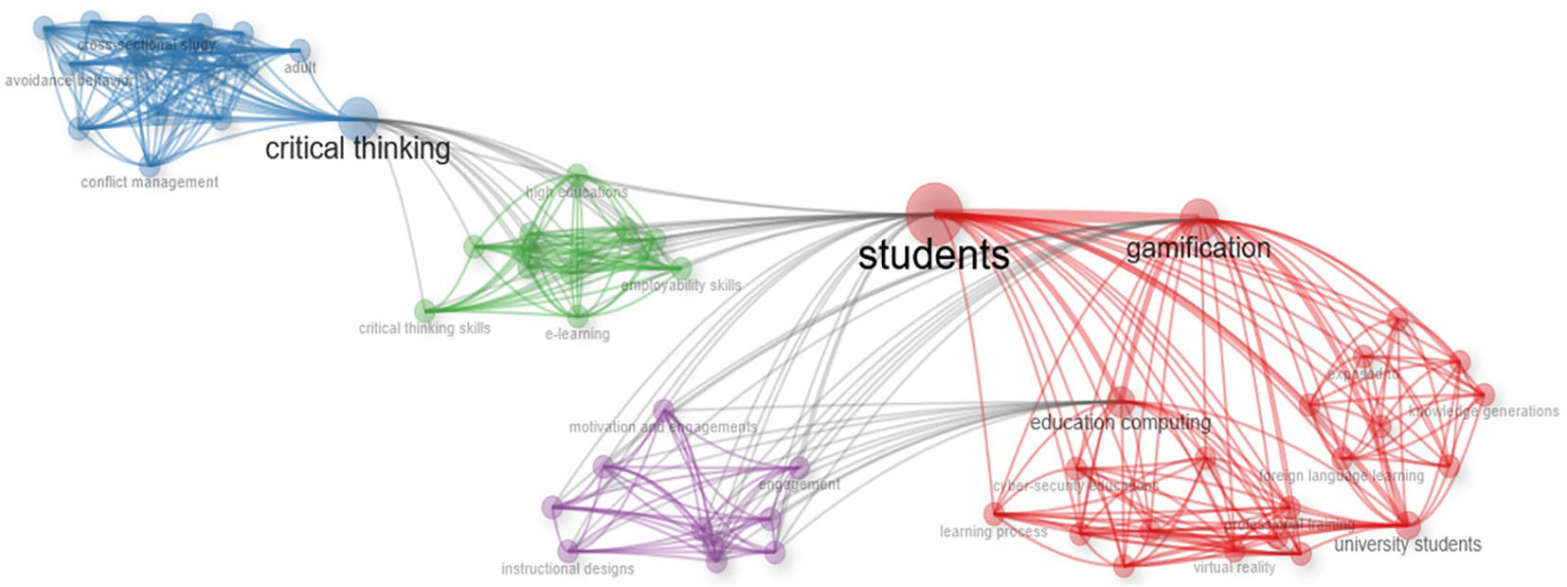
Network based on the thematic map of scientific papers extracted from SCOPUS. Note. The graph presents the network based on the thematic map of scientific papers extracted from SCOPUS.

Finally, in the case of the articles selected from the Scielo database, an interesting orientation of the studies towards the application of gamification in virtual learning environments is identified ( Adam and Berg, 2022; Medel-San Elías et al., 2023). This is consistent with Figure 13, specifically with the green quadrant where the word “e-learning” is shown, i.e., both in Scopus and Scielo there is a research area of interest on gamification in virtual learning environments.

## 5. Conclusions

The PRISMA methodology has made it possible to systematize and organize the information collected. One of the filters for the selection of documents was the last 3 years (2022, 2023 and 2024), in which articles of sufficient quality and relevance were identified to carry out an exhaustive systematic literature review. The databases used (Scopus, WOS and Scielo) are the most representative in the scientific world. The final process allowed the identification of 124 articles that were used for the analysis of this document.

The descriptive results reveal that 76% of the articles are in English and 24% in Spanish. The most prominent methodological approach is quantitative with 71%, surpassing 19% mixed, 8% reviews and meta-analysis, and 2% qualitative. The year with the highest production up to the date of data collection for this article was 2023, with 57 documents. The database that contributed the highest percentage was SCOPUS with 61%, followed by WOS with 23% and Scielo with 16%. According to the distribution of articles by study population, 92% corresponded to university students, 6% to higher education educators and 2% to studies that consider both populations.

The analysis of theoretical structures reveals that the most relevant approaches for the study of gamification are self-determination and gamified learning. It is also evident that there is a positive relationship between gamification and motivation in learning processes, which allows encouraging the development of critical thinking and the improvement of academic performance.

For future studies, it is suggested to conduct qualitative and mixed research that considers the perspective of educators in the results. Likewise, further research should be conducted on the relationship between gamification and critical thinking, where studies are still insufficient.

Research on the use of gamification presents several limitations that must be considered when interpreting results and when designing and implementing strategies based on this active methodology. Among them, the context stands out since many studies focus on specific situations, making it difficult to generalize the results to other environments. In addition, the gamification effectiveness can vary depending on the type of task, the population studied and the cultural context.

## Institutional review board statement

Not applicable.

## Registration and protocol

The protocol is detailed in the methodology section of this document.

## Data Availability

The original contributions presented in this study are included in the article. The PRISMA checklist can be found in the ZENODO repository, under the title “PRISMA flowchart and checklist - Analysis of research on gamification, critical thinking and academic performance: A systematic review in the context of higher education” ( Gamarra-Vargas et al., 2025), https://doi.org/10.5281/zenodo.16955728, License “Creative Commons Attribution 4.0 International”. Further inquiries can be directed to the corresponding author. Data are available under the terms of the Creative Commons Attribution 4.0 International license (CC-BY 4.0).
