# Supplementary material for: Analysis of research on gamification, critical thinking and academic performance: A systematic review in the context of higher education
Source: F1000Res. 2026 Feb 25;14:913. Originally published 2025 Sep 12. [Version 2] doi: 10.12688/f1000research.164232.2 (PMC13047369; doi:10.12688/f1000research.164232.2)
Supplement: Peer review history

# Reviewer report: Reviewer response for version 2

**DOI:** 10.5256/f1000research.195968.r472501

**Published:** 2026-04-10

**Authors:** John Mark Navarette Saldivar

**Affiliations:** 1 La Salle University, Ozamiz, Philippines

The manuscript addresses a timely and relevant topic and shows promise in examining the relationship among gamification, critical thinking, and academic performance in higher education. Conceptually, it identifies key constructs clearly, but their definitions and relationships remain broad and insufficiently interrogated. The theoretical grounding is present but weakly integrated, as multiple frameworks are cited without being meaningfully connected to the analysis or interpretation of findings. Methodologically, the study benefits from the use of the PRISMA framework, clear inclusion criteria, and a substantial sample size; however, rigor is limited by the absence of formal quality appraisal, insufficient explanation of coding reliability, and a largely descriptive rather than analytical synthesis. The results and discussion rely heavily on frequency counts and visual mapping, with limited critical interpretation. In terms of language and structure, the manuscript is generally understandable but requires improvement in grammar, conciseness, and paragraph organization, as redundancy and overly long sentences reduce clarity. In general, the study adds useful information, but it needs better conceptual integration, theoretical alignment, methodological depth, and language refinement. Major revision is required before the manuscript can be considered for indexing.

Are the rationale for, and objectives of, the Systematic Review clearly stated?

Yes

Is the statistical analysis and its interpretation appropriate?

Partly

If this is a Living Systematic Review, is the ‘living’ method appropriate and is the search schedule clearly defined and justified? (‘Living Systematic Review’ or a variation of this term should be included in the title.)

Not applicable

Are sufficient details of the methods and analysis provided to allow replication by others?

Partly

Are the conclusions drawn adequately supported by the results presented in the review?

Partly

Reviewer Expertise:

Social Sciences, Psychology, Language Education, Educational Technology

I confirm that I have read this submission and believe that I have an appropriate level of expertise to confirm that it is of an acceptable scientific standard, however I have significant reservations, as outlined above.

# Reviewer report: Reviewer response for version 2

**DOI:** 10.5256/f1000research.195968.r414252

**Published:** 2026-04-02

**Authors:** Ivana Roncevic

**Affiliations:** 1 University of Slavonski Brod, Croatia, Croatia

Summary

 This study is a systematic review of research on gamification, critical thinking, and academic performance in higher education. The corpus consists of studies published in Scopus, WoS, and Scielo between 2022-2024. PRISMA methodology was applied and the final corpus consisted of 124 articles. Descriptive findings document the distribution of studies by language, methodology, database, year, and population. The thematic and bibliometric analyses were conducted using Bibliometrix on a separate broader Scopus sample. Self-determination theory and gamified learning were identified as the dominant theoretical frameworks, and a positive relationship was confirmed between gamification, motivation, critical thinking, and academic performance.

 Strengths

 The authors provided a good conceptual framing and literature review. They situated gamification within a relevant theoretical landscape and cited established frameworks. The research question and the three objectives are explicitly stated. The progression from broad contextualisation to a focused review question is logical and transparent.The paper is methodologically strengthened with the bibliometric pre-analysis of a broader Scopus dataset. The identification and screening stages are transparently reported. The ten search equations (Table 1), three databases, date restrictions, language filters, and inclusion/exclusion criteria are clearly stated. PRISMA flowchart is provided and the PRISMA checklist has been deposited in Zenodo. The corpus is well described according to various criteria and is appropriately interpreted. The topic is relevant and the study addresses a genuine research gap by focusing at the intersection of gamification, critical thinking, and academic performance in higher education. The authors have responded constructively to  Reviewer 1 concerns.

**Suggested Changes**

 1.The study should differentiate between the two distinct datasets. The bibliometric analysis in the introduction (Figures 1–5) draws on a broader Scopus query of 712 documents on gamification (2020–2024, all open access, social sciences), while the systematic review corpus comprises 124 articles identified through a different, more restrictive search equation across three databases. These seem to be methodologically distinct datasets serving different purposes. If so, the article should consistently make this distinction explicit and maintain it until the end.

 2. Quantitative validation of network claims. In the revised version, the qualitative discussion of network centrality should be supported with quantitative results. As Reviewer 1 noted, degree centrality and betweenness centrality values should be reported for key nodes in Figures 12 and 13. This would substantially strengthen the interpretive claims made in the discussion.

 3. Thematic coding. In the methods section of the revised paper, the inductive thematic coding process is described. Advised is the report of  intercoder reliability or agreement statistics. Given that all five authors served as independent reviewers and discrepancies were resolved by consensus, some quantitative measure of agreement (e.g., Cohen's kappa or percentage agreement at an intermediate coding stage) would demonstrate procedural rigour and reduce the risk of interpretive bias.

 4. Given that the search was conducted in August 2024, the 2024 figure necessarily represents a partial year, and this artefact of the data collection timing should be explicitly acknowledged as the most probable explanation for the apparent decline in publications captured in the corpus.

 5. The reviewer suggests a more detailed insight into the near-absence of qualitative methodology in the studies included in the corpus. This near-absence of qualitative work represents a substantive research gap that deserves more analytical attention and may be addressed in limitations or future research agenda.

 6. The thematic analysis is largely based on studies in Scopus and WoS, even though 16% of the corpus is comprised from Scielo-indexed studies. Those are addressed only briefly in the final paragraph of the discussion. This is relative marginalisation in the thematic analysis and can be explained as a limitation that should be acknowledged more explicitly. The authors may consider whether any meaningful comparison between regional and global publication trends is possible.

 7. Table 2 lists selected articles by database, but some articles appear as duplicates. It should be explained how duplicates were handled in the final coding.

Are the rationale for, and objectives of, the Systematic Review clearly stated?

Yes

Is the statistical analysis and its interpretation appropriate?

Not applicable

If this is a Living Systematic Review, is the ‘living’ method appropriate and is the search schedule clearly defined and justified? (‘Living Systematic Review’ or a variation of this term should be included in the title.)

Not applicable

Are sufficient details of the methods and analysis provided to allow replication by others?

Partly

Are the conclusions drawn adequately supported by the results presented in the review?

Partly

Reviewer Expertise:

Applied Linguistics, E-learning, Education Technology, Discourse Analysis, Digital Rhetoric

I confirm that I have read this submission and believe that I have an appropriate level of expertise to confirm that it is of an acceptable scientific standard, however I have significant reservations, as outlined above.

# Reviewer report: Reviewer response for version 1

**DOI:** 10.5256/f1000research.180706.r414254

**Published:** 2025-10-10

**Authors:** Rafael Mellado S.

**Affiliations:** 1 Pontificia Universidad Católica de Valparaíso, Valparaíso, Chile

This article is a systematic review analyzing research on the relationship between gamification, critical thinking, and academic performance in higher education. Using the PRISMA methodology, the authors searched the Scopus, WoS, and Scielo databases, limiting the search period to the last three years (2022–2024). The final corpus included 124 articles.

 The descriptive results reveal a strong predominance of English-language literature (76%) and a clear bias toward quantitative methodology (71%). The majority of studies overwhelmingly focus on university students (92%). In-depth thematic analysis indicates that Self-Determination Theory and Gamified Learning are the most relevant theoretical approaches. The main findings confirm a positive relationship between gamification and learning motivation, which in turn is linked to fostering critical thinking and improving academic performance.

 The replicability of the article identification and screening phase is high: the databases, search dates, inclusion/exclusion criteria (e.g., only undergraduate students and articles from 2022–2024) are named, and the 10 exact search equations (Table 1) and the PRISMA flowchart are provided.

 Replicability fails in the content analysis and data visualization phase. For the network analysis (Figures 12 and 13) to be reproducible, the authors should:

 1. Detail Software Parameters: Specify in the Methods section the exact parameters used to generate the bibliometric and thematic maps (Figures 3, 4, 5, 12, 13). This includes the name of the software (if different from Bibliometrix), the clustering algorithm (e.g., modularity), and the minimum thresholds (e.g., minimum number of occurrences or citations) used to include terms or nodes in the visualization.

 2. Data Extraction Protocol (Data Charting): Given that the review was manual, the protocol for data extraction and coding of the thematic findings, which served as the basis for the conceptual networks, should be described in greater detail.

 The analysis is suitable for a descriptive study or systematic mapping. The distribution data (language, methodology, year, databases, and population) are clearly presented and graphed.

 The network analysis (Discussion) is qualitative in nature, which reduces the rigor of the interpretation of the importance of the themes. To improve robustness:

 1. Quantitative Network Validation: The interpretation that certain clusters or nodes are "central" should be supported by centrality metrics. The authors should integrate and report Degree Centrality or Betweenness Centrality values ​​for key nodes (gamification, motivation, critical thinking) that numerically demonstrate their importance as "bridges" or their number of connections within the network. This would transform the visual interpretation into a scientifically quantifiable conclusion.

Are the rationale for, and objectives of, the Systematic Review clearly stated?

Yes

Is the statistical analysis and its interpretation appropriate?

Partly

If this is a Living Systematic Review, is the ‘living’ method appropriate and is the search schedule clearly defined and justified? (‘Living Systematic Review’ or a variation of this term should be included in the title.)

Not applicable

Are sufficient details of the methods and analysis provided to allow replication by others?

Partly

Are the conclusions drawn adequately supported by the results presented in the review?

Yes

Reviewer Expertise:

NA

I confirm that I have read this submission and believe that I have an appropriate level of expertise to confirm that it is of an acceptable scientific standard, however I have significant reservations, as outlined above.

# Response

**Published:** 2026-01-29

**Authors:** NATALIE GAMARRA

**Affiliations:** Lima, Peruvian University of Applied Sciences, Lima District, Lima Region, Peru

Dear Reviewer Rafael Mellado,

 Thank you very much for the time and effort you devoted to reviewing our article. We truly appreciate your valuable comments and suggestions.

 In response to your observations, we have incorporated the following changes and improvements into the manuscript:

1. We have included a clearer explanation of the use of the Bibliometrix software for processing the review data.
2. We have added a more detailed description of the data extraction process used in the study.
3. We have strengthened the analysis of the networks and nodes presented in Figures 4, 12, and 13. Additionally, the quantitative analysis is presented in Figures 7, 8, 9, 10, and 11. Therefore, the article now integrates both quantitative and qualitative analyses, providing a more comprehensive interpretation of the results.

Thank you again for your constructive feedback, which has significantly contributed to improving the quality of our work.

 Kind regards,

 Natalie Gamarra

# Reviewer report: Reviewer response for version 1

**DOI:** 10.5256/f1000research.180706.r414255

**Published:** 2025-09-18

**Authors:** Angélica Inês Miotto

**Affiliations:** 1 IESGO, Goiás, Brazil; 2 University of Aveiro, Aveiro, Portugal

The methodological design of the study combines well-described, solid, detailed, and consistent methods and sources. In this sense, the results and discussions explored encourage researchers to engage in new reflections, as well as to generate knowledge using other tools. It is also observed that efforts are directed toward the field of gamification, critical thinking, and academic performance in higher education, demonstrating potential for creative processes and the pursuit of idea generation.

Are the rationale for, and objectives of, the Systematic Review clearly stated?

Yes

Is the statistical analysis and its interpretation appropriate?

Yes

If this is a Living Systematic Review, is the ‘living’ method appropriate and is the search schedule clearly defined and justified? (‘Living Systematic Review’ or a variation of this term should be included in the title.)

Yes

Are sufficient details of the methods and analysis provided to allow replication by others?

Yes

Are the conclusions drawn adequately supported by the results presented in the review?

Yes

Reviewer Expertise:

Education and Education Policies

I confirm that I have read this submission and believe that I have an appropriate level of expertise to confirm that it is of an acceptable scientific standard.
